# Characterization of Three Amu-Darya Basin Clays in Ceramic Brick Industry and Their Applications with Brick Waste

**DOI:** 10.3390/ma14237471

**Published:** 2021-12-06

**Authors:** Serdar Korpayev, Meretdurdy Bayramov, Serdar Durdyev, Hemra Hamrayev

**Affiliations:** 1 Economic Society “Dowletli-Dowran”, Halach District, Lebap Velayat, Khalach 746632, Turkmenistan; meretdurdyb@gmail.com; 2 Department of Chemistry, Hacettepe University, Beytepe, Ankara 06800, Turkey; 3 Department of Engineering and Architectural Studies, Ara Institute of Canterbury, 130 Madras Street, Christchurch 8011, New Zealand; 4 Malaysia-Japan International Institute of Technology, Universiti Teknologi Malaysia, Jalan Sultan Yahya Petra, Kuala Lumpur 54100, Malaysia; hhamrayev@gmail.com

**Keywords:** clays, Amu-Darya basin, ceramic brick, characterization, building industry

## Abstract

This study examined the chemical, mineralogical, physical, thermal, and technological characteristics of the Dostluk (DM), Halach (HM), and Sakar (HM) clay deposits located in the Amu-Darya basin of Turkmenistan. The potential suitability of these deposits was evaluated for the local ceramic brick industry. The chemical and mineralogical features were identified by X-ray fluorescence (XRF), ion chromatography (IC), energy-dispersive X-ray spectroscopy (EDS), and X-ray diffraction (XRD) techniques. The physical properties were characterized by granulometric analysis by sieving, particle size distribution, scanning electron microscopy/optic analysis, specific surface area, Pfefferkon’s plasticity index, reabsorption, shrinkage, water absorption, mechanical (compression and bending), and freeze–thaw durability tests. The thermal methods were performed using dilatometry and thermogravimetric/differential thermal analyzer (TG/DTA). The test samples for the different clay deposits were extruded, dried, and fired at three different temperatures of 850 °C, 950 °C, and 1050 °C. While the Dostluk and Sakar clays have high plasticity, Halach clay has been found to have low plasticity. The mechanical and freeze–thaw durability tests demonstrated that the outcomes of the clays of different origins were sufficient, achieving compressive strengths of over 10 MPa and mass loss less than 3%, which are acceptable by industry standards. Semi-industrial processed hollow bricks demonstrated promising characteristics. While the Dostluk and Sakar clay-based brick specimens were visibly free of cracks, the Halach specimens showed some cracks. The physical and mechanical improvements of these clays were performed with three mixtures, which are M1 (80 mass% DM + 20 mass% brick waste), M2 (85 mass% SM + 15 mass% brick waste), and M3 (70 mass% HM + 25 mass% SM and 5 mass% brick waste) for the brick industry.

## 1. Introduction

The building industry has an important role in the economic development of developing countries as it is directly related to many industries, such as cement, concrete, steel and other metals, glass, tile, and bricks [1]. One of the most important of these sectors is the brick industry, which has a global production of approximately 1.391 trillion pieces in 2014 annually [2]. 

Clays are among the most frequently used and versatile materials in industrial fields, such as the petroleum industry; they are also used in the composition of foundry molds, paint, paper coatings and fillings, pharmaceuticals, and water treatments (catalysis, adsorbents, ion exchangers) [3,4,5,6]. Clays are also essential components of ceramic products and building materials [7]. It is important to understand the features of clays for diverse industrial applications. The features of clay materials are chiefly determined by their chemical and mineral composition [8], organic content, particle size distribution, plasticity [9], and moisture [10]. The technological treatment of clay materials is also important. This includes forming technology, applying pressure, drying and firing processes, and soaking time, etc. [11].

There are two main types of bricks—cement-based and clay-based bricks [12]. Clay-based bricks have many advantages compared with cement-based bricks: they are cheap, environmentally friendly, safe for human use, require less energy to produce, and have high durability and fire resistance [13,14,15]. Furthermore, clay-based bricks are an excellent building material for both human use and the environment [16]. This was also confirmed by their green building credits in rating programs, such as Green Star and Leadership in Energy and Environmental Design (LEED), which reflect their important environmental contribution [17]. Kiln firing makes these clay-based bricks extremely tough and stiff. The clay-based bricks have advantages to balance the home’s thermal mass and heat. They often do this by storing and absorbing heat by intelligently making effective active or passive heating systems; in other words, they can control internal temperatures as well as provide additional noise insulation [18]. When clay-based bricks are used externally, they provide superior protection against extreme weather conditions, especially dangerous wind-blown debris.

Given these advantages, the exploration of new clay deposits in different regions of the world is important to meet the high demand for clay-based bricks from the renewable and green building industry. The Amu-Darya River is the largest river basin in Central Asia. Rising in the Pamir Mountains, it is formed by the confluence of the Vakhsh, Panj, and Kunduz Rivers. The Amu-Darya basin in Turkmenistan was studied by Brunet et al. (2017), who used geological and geophysical data to reconstruct the Late Palaeozoic and Mesozoic evolution [19]. According to Brunet et al. (2017), the major tectonic events in the formation and evolution of the Amu-Darya basin occurred in three steps: (1) Late Palaeozoic to Early Triassic, (2) Middle Triassic to the Triassic–Jurassic boundary, and (3) Early to Middle Jurassic [19]. 

Clays, which are natural resources, are constantly used and are subject to extinction with passing time. Waste brick (WB) is silicate industrial solid waste from either brick factories or the construction industry [20]. The volume of WB resulting from daily activities, ongoing construction, production, and industry continues to increase rapidly in order to meet the demands of the increasing population, and its recycling has great social and environmental importance [16]. In factories producing bricks, a significant amount of brick loss occurs due to different technical reasons, and this creates a problem for the environment in the form of waste bricks. The WB has been disposed of in landfills, causing environmental problems over the past century. The use of wastes in clay bricks has generally had positive effects on the properties with enhanced strength, shrinkage, porosity, and thermal features [20,21,22]. In general, recycling WB in fired clay bricks is practical and useful when the correct percentages are included and also functions as an alternative disposal method to potentially contaminating wastes. The brick producer will also minimize the cost of the clay materials, the utilization of energy during firing, and the improvement of the ceramic brick features.

The main objective of this study is to examine the physicochemical features and the characterization of the ceramic bodies obtained from the Dostluk, Halach, and Sakar clays. The study evaluates the suitability of these clays using the latest diverse characterization techniques. To the best of the researchers’ knowledge, this work is the first suitability assessment of illitic Dostluk mix (DM), Halach mix (HM), and Sakar mix (SM) clays with some applications to the building industry. The study examines three different clays as raw materials to contribute to the study of the mineralogical and physicochemical features of Amu-Darya basin clays for the newly established ceramic brick industry in the Lebap region of Turkmenistan. The second objective of this work is to improve the physical and mechanical performance of clay-based bricks by using WB from this factory by making mixtures at optimum WB ratios. The output of this study provides insights into the possible methods of improving the building material features of clay-based bricks by using WB at optimum ratios.

## 2. Materials and Methods

### 2.1. Sample Materials

**Figure A1:**
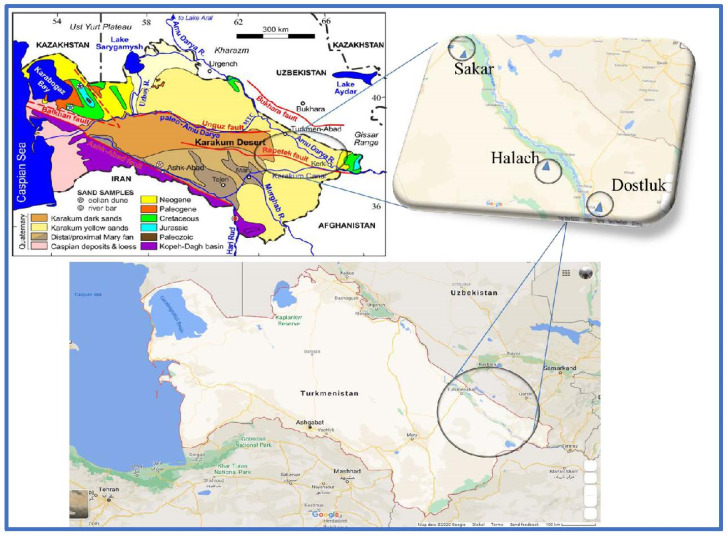
Location of Dostluk, Halach, and Sakar clays along the Amu-Darya River. The geological map image of Turkmenistan edited from the study [72].

**Figure A2:**
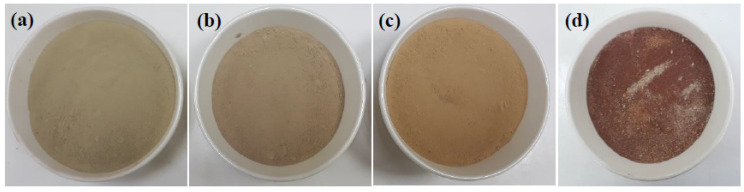
The images of (**a**) DM, (**b**) HM, (**c**) SM, and (**d**) the powder form of brick waste.

The study characterized the most suitable clays collected from three different regions of Amu-Darya basin, Turkmenistan, for the ceramic brick industry. For this purpose, deposits were selected from three distinct places, namely Dostluk, Halach, and Sakar. From a geological perspective, these three deposits are easily accessible on the edge of the Amu-Darya basin. As shown in Figure A1 (Appendix A), these three new deposits will be used in the production of bricks. The Dostluk deposits (latitude 37°48′–38°49′ N and longitude 65°24′–65°20′ E) are located 21 km southeast of the town of Kerkichi, Turkmenistan. The sedimentary series in the area of Dostluk date from the late Cretaceous era. The Sakar deposits (latitude 38°49′–38°50′ N and longitude 63°47′–63°48′ E) are located 11 km northwest of Sakar City. The sedimentary series in the Sakar region consists of upper Neogene–middle Eocene formations. The Halach deposits (latitude 38°05′–38°04′ N and longitude 64°51′–64°57′ E) are located at 19 km north of the town of Halach on the banks of the Amu-Darya River. The Halach deposit is alluvial clay from the Quaternary period of the Cenozoic Era. The used clays in the study were provided by geologists from the relevant deposits with the permission of the local government. The clay mixtures of each deposit were collected from five different regions (R1 to R5) for each deposit. The gently crushed five representative samples for each deposit are called the Dostluk mix (DM), the Halach mix (HM,) and the Sakar mix (SM). Samples were collected at intervals of 15 to 20 m and depths of 1.5 to 2.0 m. The DM, HM, and SM mixtures were a representative sample of five clay samples (no less than 50 kg of clay) selected from Dostluk, Halach, and Sakar, respectively. After collection, the clay samples were dried at 110 °C for 24 h, and then they were powdered gently by a hammer mill. The WB obtained from the factory was also powdered gently by using a hammer mill (Figure A2 (Appendix A)). 

### 2.2. Specimen Preparation, Extrusion, Drying, and Firing Process of Clay-Based Bricks

**Figure A3:**
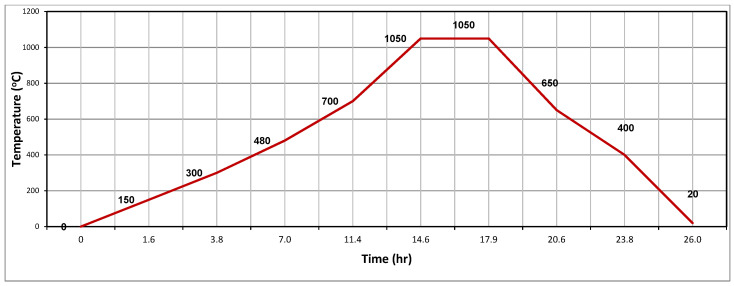
Firing cycle of DM, HM, and SM.

After pre-treatments (drying and milling), the DM, HM, and SM clays were sprayed with about 19, 17, and 18 wt% of the total mass of the samples (6000 g), respectively, and mechanically stirred for 10 min. The amount of water required to prepare mud with these clay types was found by adjusting the penetrometer values between 1.8 and 2.5 kg/cm^2^. Afterward, the humidified clays were left undisturbed in sealed plastic boxes overnight. The extrusion of the clays was carried out with a 050C extruder (Verdés, Barcelona, Spain) for specimen production in 120 × 30 × 18 mm^3^ dimensions. The extrusion parameters were 80 cm Hg of vacuum, 30 bar of pressure, 18 units/min of production capacity, and 33 °C to 39 °C of extrusion temperature at the exit of the extruder. The shaped samples were gradually dried in an oven at an increasing temperature between 30 °C and 110 °C until a constant mass was obtained in order to eliminate the free water content (TypeM40, Ceramic Instruments, Sassuolo, Italy). Drying time and process varied according to the nature of the clays. If the sensitivity values obtained from Bigot’s curve are greater than 2, the wet bricks should be dried carefully. Following the drying, the specimens were finally fired at 850 °C, 950 °C, and 1050 °C within a firing cycle of 26 h. The firing cycle of DM, HM, and SM clay-based is given in Figure A3 at a heating rate of 1.3 °C/min. This included heating and cooling in an electrical laboratory chamber Nabertherm oven (Siemens, Bremen, Germany). 

### 2.3. Preparation of Clay and WB Mixtures for Brick Production

The powdered clays of DM and SM were mixed with WB to make mixtures, which are M1 (80 mass% DM + 20 mass% WB) and M2 (85 mass% SM + 15 mass% WB), respectively. The mixture of HM with SM and WB clay was M3 (70 mass% HM + 25 mass% SM + 5 mass% WB). After preparing these compositions, the same procedure was followed, which was described in Section 2.2. The waste bricks that were ground into powder are shown in Figure A2d (Appendix A).

### 2.4. Evaluation of Technological Parameters of the Specimens

The technological features of the extruded pieces were determined according to established ceramic procedures. To determine the contents of the soluble salt of DM, HM, and SM, 50 gr of clay was weighed and 500 mL of boiled water was added, stirred, and filtered. The concentration of soluble salts was detected using a Dionex™ ICS-5000+ Capillary HPIC (Thermo Scientific, Waltham, Massachusetts, USA). The calcium carbonate content of clays was determined using the Volumetric Calcimeter Method (Soil Calcium Carbonate Equivalent) by using calcimeter apparatus. For this, carbonates were treated with hydrochloric acid (Equations (1) and (2)) and the volume of carbon dioxide released was measured. At constant pressure and temperature, as a result of clay carbonate decomposition, the change in water level in the system is a direct measure of the resulting CO_2_ mass. Calcium carbonate (CaCO_3_) equivalent was measured as follows (Equation (1)):

(1) $${\mathrm{CaCO}}_{3}\;\mathrm{equiv}.,\;\%=(\frac{M_{{\mathrm{CaCO}}_{3}}}{M_{\mathrm{clay}}})\;\times \;100$$

where M_CaCO3_ = mass of CaCO_3_ calculated from the calibration curve (g), M_clay_ = mass of clay (g). 

The moisture content was measured using two techniques: by drying at 110 °C until a constant mass was reached, and by placing 0.01 g of the clay mixture into an MA 50R Moisture Analyzer (Radwag, Radom, Poland). The mass loss on drying and firing was determined by weighing the specimens. The loss on ignition (LOI %) of the specimens was determined by measuring their mass changes before and after the firing at 1000 °C (Equation (2)).

(2) $$LOI\%=\frac{M_{d}-M_{f}}{M_{d}}\times 100$$

where M_d_ = mass of oven-dried specimens (g) at 110 °C and *M_f_* = mass of fired specimens (g) at 1000 °C. Linear drying and firing shrinkages of the specimens were determined with an indent marker stage following the test standard method *ASTM C210-95*. Reabsorption and water absorption were calculated based on *ASTM C373-88* standard [23]. For reabsorption experiments, the specimens were weighed before and after being placed into the humidified atmosphere. Sieve analysis of grain size was performed to determine individual grains of sediment by using the BS test sieves: 1000 g of clay samples were weighed, dissolved in water, and poured into sieves of different sizes on a mechanical shaker BA 200N (CISA, Barcelona, Spain) and shaken for 15 min. The mass of each sieve was determined after being dried in the oven at 100 °C. The percent retained (PR) after passing through each sieve was calculated using (Equation (3)).

(3) $$P_{R}=\frac{\mathrm{Mass}\;\mathrm{retained}\;\left(M_{R}\right)}{\mathrm{Initial}\;\mathrm{mass}\;\left(M_{i}\right)}\;\times \;100$$

Plasticity (Ps) is a significant parameter to control extrusion failures and heterogeneities that is used to characterize clay deformation [24]. The plasticity (Ps) was determined using (Equation (4)).

(4) $$P_{s}=\frac{m_{w}\;-{\;m}_{d}}{m_{d}}\times 100$$

where m_w_ = wet state mass of the sample (g) and m_d_ = mass of oven-dried specimens (g). The physical behaviors of fired brick specimens, such as bulk density, apparent porosity, and apparent specific (AS) gravity, were determined according to *ASTM C373-88* standard [23]. 

### 2.5. Characterization of Clays and Bricks 

The mineralogical characterization of DM, HM, and SM clays as raw and fired materials was determined by powder X-ray diffraction (XRD). The XRD analysis was carried out at standard conditions (40 kV, 30 mA, 0–80° 2θ, a step size of 0.017°, and a dwell time of 10 s step^−1^) with Rigaku Ultima IV diffractometer equipped with a Cu-Kα1 radiation source (λ = 1.5406 Å). Before the analysis, the sample preparation was performed by following the criteria expressed in Moore and Reynolds (1997) [25]. The oriented aggregates of three clays were subjected to three different successive treatments: air drying, glycolation, and heating to 550 °C for 2 h to confirm the type of clay mineral phases. The chemical composition of the clays, particularly for the major oxides, was determined using an X-ray fluorescence (XRF) spectrometer (Bruker S4 Pioneer, Karlsruhe, Germany). XRF measurements of powder clays (<63 µm) were carried out following BSEN ISO 12677:2013, which was operated at 0.8 mA, 40 kV, and 134.7 eV spectrum resolution under vacuum. For the semi-quantitative analysis, XRF was calibrated with a blank specimen and commercial air filter standards from Micromatter (Vancouver, Canada). The used standards for calibration were fabricated pure elements and oxides deposited on 37 mm nucleopore filters. The specimen air filters were placed in a tailored stainless steel air filter specimen holder along with an air filter monitor, an X-ray monitor, and the filter blank and were examined utilizing an automated set. It was utilized to correct the data for drift or variations of medium and long term in the XRF response. The particle size distribution and mean particle size of the clays were analyzed using the Mastersizer Hydro 3000E (Malvern, UK). The specific surface area of clays was obtained from the particle size distribution using the laser diffraction technique (ISO 13320-1). The morphological and elemental characterization of the clays, unfired bricks, and fired bricks made from DM, HM, and SM clays were determined using field emission scanning electron microscopy equipped with an EDAX Energy Dispersive X-ray Analyzer (SEM, JEOL JSM-5800, Tokyo, Japan) at an operating voltage of 20 kV and a working distance of 10 mm. Before the SEM analysis, the clay and brick specimens were dried and coated with platinum in a vacuum to increase the conductivity of samples. The thermal behavior of clays was determined using a Thermo-Gravimetric/Differential Thermal Analyzer (TG/DTA: Seiko EXSTAR6000, Chiba, Japan). The temperature was increased from room temperature to 1200 °C at a rate of 2 °C/min with a nitrogen flow. Bending strength analysis of the clay-based bricks (~105 mm × 28 mm) was performed at 0.5 mm/min loading with a mechanical test instrument (Ibertest, Spain) with a test of 100 kN capacity. The compressive strength tests of brick specimens (120 mm × 250 mm) were carried out with 4000 kN hydraulic universal testing machine (Besmak, Ankara, Turkey) at a loading rate of 0.5 kN/s following ASTM C67-03 [26].

### 2.6. Evaluation of Plasticity

The suitability of the clay materials for clay-based bricks was evaluated by using the Pfefferkorn method, which is based on the principle of impact deformation [9,27]. This method, described by Amorós et al., was used to determine Pfefferkon’s plasticity index (PPI) [28]. This method calculates the amount of water needed to achieve a 30% contraction of the initial height (H_0_) of a test specimen under the action of a standard mass. The PPI value was obtained from Pfefferkorn straight lines of moisture content% vs. height ratio of the specimen [9,29]. Then, the plasticity and consistency of the rods were evaluated using a pocket soil penetrometer ST207 (kg/cm^2^) based on penetration. The standards for the penetrometer were BS 1377 (1990) [9]. Extrusion measurements with a penetrometer are classified as soft (1.2–1.8 kg/cm^2^) and stiff (3–4.5 kg/cm^2^), with the preferred range for consistency being 1.8–3 kg/cm^2^ [30].

### 2.7. Bigot’s Curves

Bigot’s curves are typically used as a routine control in custom clay-based brick production to test the sensitivity of clays and specimens to drying. Bigot’s curves indicate the evolution of the linear shrinkage of specimen moisture In brief, the initial point is the highest moisture content at the beginning of the drying process. The linear shrinkage occurs as a result of water evaporating from the structure of the specimens [30]. Bigot’s curves were drawn using an Adamel barelattograph to characterize the drying process of the specimens (200 mm × 80 mm × 9 mm extruded rods). This measures the changes in length and shape of a slide within 48 h by adding water to the raw material until a normal paste is produced that does not stick to fingers. While a sample was fixed to the apparatus to record the curve, the other sample was used to calibrate the weighing system of the apparatus at several time intervals. After the sample had finished shrinking, it was dried at 110 °C. The length (l) and mass (m) of each slide were recorded before and after complete drying. This technique determines the critical point that graphically separates the two drying phases. In the first phase (colloidal water), the sample shrinks as it gives off the water; in the second phase (interposition water), the external dimensions of the sample remain nearly constant despite continued water extraction. The coefficients of sensitivity to drying by Bigot (CSB) values are classified as insensitive (<1), medium sensitive (1.0–1.5), sensitive (1.5–2.0), and high sensitive (>2.0).

### 2.8. Dilatometer Test

The firing characteristics of the DM, HM, and SM clays were determined by heating the sample up to 1100 °C using an Expedis DIL 402 Classic Dilatometer (NETZSCH, Selb, Germany). The cylindrical sample (25 × 6 mm) was pressed from the humidified powder and dried overnight at 40 °C, then placed in a horizontal expansion dilatometer and heated between 25 °C and 1100 °C at a heating rate of 10 °C/min. During the heating stage, the length changes were recorded per minute. The thermal expansion coefficients of clays were determined using dilatometric measurements within a certain temperature interval. The linear thermal expansion coefficient is expressed by the following formula:

(5) $$\alpha_{T_{1}-T_{2}}=\frac{\Delta L}{\left(L_{0}\times \Delta T\right)}$$

where *α* is the linear thermal expansion coefficient between *T*_1_ and *T*_2_, Δ*L* is the difference between *L_T_*_1_ and *L_T_*_2_, and *L* is the initial length of the specimen. 

### 2.9. Frost Resistance of Clay-Based Bricks

To evaluate the frost resistance of the brick specimens against freeze and thaw (freeze–thaw durability), the specimens were placed in freezing and thawing conditions according to the *ASTM C67* standard test method using DFR/60 (Ceramic Instruments, Italy). After impregnating the samples with deionized water, the brick specimens were submitted to a cycle of between +5 °C and −5 °C and were kept in freezing and thawing conditions for 200 cycles. Each cycle was kept for 15 min under zero and 15 min submerged in water at a temperature above +5 °C for thawing purposes. The brick samples were visually monitored daily for cracks and other damage. Last, the specimens were weighed to calculate mass loss% after 200 cycles, and the outcome was given as %mass loss. Further, the initial water absorption (E_1_) and final water absorption (E_2_) were determined after the freeze–thaw cycles.

## 3. Results and Discussion

### 3.1. Sample Material

**Figure 1:**
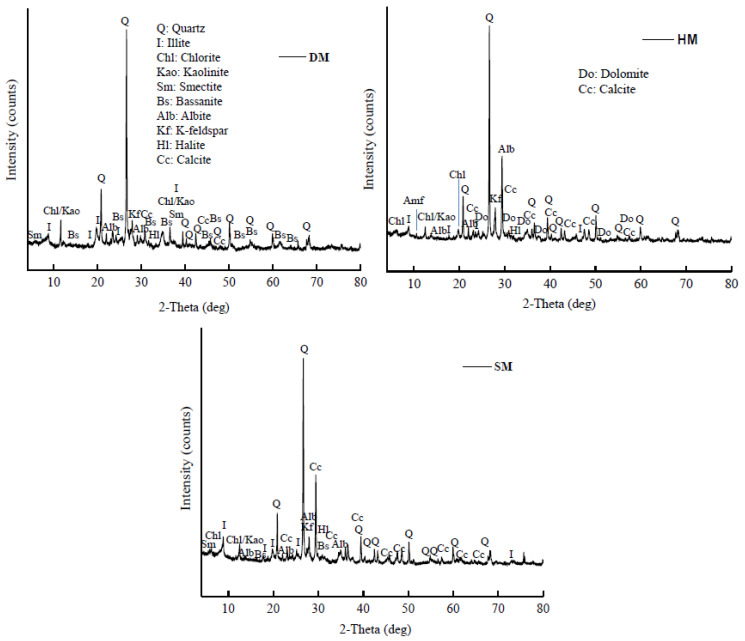
XRD spectrum of the glycolated DM, HM, and SM clays.

Upon arrival, the DM, HM, and SM clays were in block-sized portions > 5 cm with 4.20%, 0.80%, and 2.60% moisture values, respectively. These clays from different deposits were distinguished by their colors (Figure 1). While the DM and HM clays were greyish, the SM clay was red (Figure A2). After granulation by Hammermill, 98 wt.% of the dry sample was smaller than 500 μm in all the clays. The small amount of calcium carbonate (CaCO_3_) particles found in HM and SM were greater than the 500 μm sieve portion, which led to the formation of the free CaO phase (white grains) in the fired brick specimens. Table 1 shows the wet sieving and carbonate determination test outcomes of the DM, HM, and SM clays for brick production. The carbonate content of clays is important because it can only be up to 15% for clay suitability for brick production [31]. The outcomes of the sieve analysis performed on the DM, HM, and SM clay samples demonstrated that 94.43% of DM, 93.8% of HM, and 86.71% of SM, respectively, pass through 80 μm. For DM, HM, and SM, wet sieving demonstrated a very fine particle size distribution with a residual fraction of 1.51%, 39.25%, and 8.55% on 63µm, respectively. Thus, HM clay is mainly composed of quartz in the residue, whereas some portions of quartz and mica were observed in DM and SM. The chemical analysis shows the presence of total carbon in the quantity of 0.12%, 2.08%, and 1.73% for DM, HM, and SM, respectively. The calcium carbonate contents of DM, HM, and SM were 1 to 1.5%, 10.50%, and 12.90%, respectively. The total soluble salt content and concentrations (ppm) of some significant ions (Na^+^ + K^+^, Mg^2+^, Ca^2+^, SO_4_^2−^, Cl^−^, HCO_3_^−^, and CO_3_^2−^) in these clay types are provided as an appendix (Table A1, Appendix A). The highest total salt content (%) was observed in DM with a value of 0.42%, which is less than 0.5% by mass as a threshold value for brick production [32]. The pH values were 7.32, 7.80, and 6.82 for DM, HM, and SM, respectively (Table A1). The percentage of pollutant elements (sulfur) for DM and SM were 0.3% (S) and 0.07% (S), respectively, which were considered very low levels. The emission of these gases into the environment during the mass production of bricks after firing is a serious problem for the environment and human health [33]. The formation of pores in DM, HM, and SM clay-based brick specimens was predicted based on their chemical composition (high amount of carbonates) and salt content.

### 3.2. Chemical Composition (Oxide Content %) of the Raw Materials

The major components of the DM, HM, and SM clays based on the chemical composition characterization with XRF are summarized in Table 2. While DM clay contains silica (SiO_2_) 57.39%, alumina (Al_2_O_3_) 16.90%, iron (III) oxide (Fe_2_O_3_) 6.28%, magnesia (MgO) 2.23%, potassium oxide (K_2_O) 1.76%, sodium oxide (Na_2_O) 1.87%, quicklime (CaO) 2.15%, and traces of MnO, P_2_O_3_, and TiO_2_, HM clay contains SiO_2_ 50.20%, Al_2_O_3_ 14.70%, Fe_2_O_3_ 2.80, MgO 2.47%, K_2_O 1.76%, Na_2_O 2.0%, CaO 12.70%, and traces of MnO and P_2_O_3_. SM contains SiO_2_ 53.09%, Al_2_O_3_ 11.86%, Fe_2_O_3_ 5.55%, MgO 2.35%, K_2_O 2.91%, Na_2_O 2.21%, CaO 8.55%, and traces of MnO and P_2_O_3_.

The high values of LOI, especially in the HM and SM clays (with 12.40% and 12.31% values, respectively), could be attributed to the presence of combustible species, such as organic matter and carbonates. The LOI value of DM clay was 7.15%, which is lower than the HM and SM clays (Table 2). The gypsum content of DM clay also has an effect on the obtention of the LOI value. The higher LOI values of HM and SM were due to the higher amounts of organic materials and carbonates in their structures. Generally, clays used in brick manufacturing should be characterized by a ratio ranging from 0.5 < SiO_2_/Al_2_O_3_ < 4.5 [34]. The molar ratios of SiO_2_/Al_2_O_3_ for DM, HM, and SM were 1.64, 1.63, and 1.29, respectively, showing their suitability for brick production. The SiO_2_/Al_2_O_3_ values of 4.5 and 0.5 indicate excess SiO_2_ and Al_2_O_3_, respectively. The HM and SM clays are considered calcareous because of their considerable CaO content, which is higher than 6% [35]. Further, the synergetic and combined effect of Fe_2_O_3_, MgO, K_2_O, and Na_2_O, also known as flux agents, was higher than 9%, except for HM (8.90%), suggesting promising suitability as a raw material. This effect also provides the development of glassy material, which stiffens the fitting of constituents by binding the crystalline minerals [36,37]. 

### 3.3. Mineralogy of the Raw Materials

The mineralogical characterization of the DM, HM, and SM clays as raw materials is shown in Table 2. The XRD outcomes demonstrate that the DM, HM, and SM clays used in clay-based brick production were rich in illite (I), with a considerable amount of quartz (Q), K-feldspar, and albite (Alb) (Figure 1 and Table 2). The clays also contained smectite (Sm), chlorite (Chl), calcite, and dolomite (Dol) in small amounts. The albite or sodium feldspar (NaAlSi_3_O_8_) were found in all the clays. In contrast, dolomite and calcite were not seen in the DM clays; the main components of this clay were illite and kaolinite. Bassanite or gypsum (CaSO_4_·2H_2_O) was also present in the DM clays, as well as traces of smectite (Sm), albite (Alb), and halite (Hl, NaCl). While the HM and SM clays showed the presence of dolomite (CaMg(CO_3_)_2_) and calcite (CaCO_3_), the DM clay did not show the dolomite mineral and showed traces of calcite. The dolomite minerals are known to be flame-retardant or flame-resistant because of their magnesium constituents.

Accordingly, the DM clay-based bricks melted at 1100 °C because of the absence of dolomite and calcite minerals and higher amounts of fluxes (Na_2_O, K_2_O, etc.) in their structure. The higher illite content of DM has also an effect on this outcome by forming a liquid phase at a lower temperature [38]. Moreover, illite is extensively utilized as a fluxing material in the conventional ceramic industry [39]. In contrast, the HM and SM clays were resistant to firing at 1100 °C. Moreover, based on the chemical composition analysis and technological characteristics, all the clay types are likely to be mostly composed of a clay mixture (mainly of chloritic/illitic origin) with a smaller fraction of quartz. The mineralogical compositions of the clays reveal that they contain the appropriate illite, quartz, kaolinite, and feldspar content to be suitable for ceramic brick products.

### 3.4. Microgranulometric and Particle-Size Distribution Analysis of Clays

**Figure 2:**
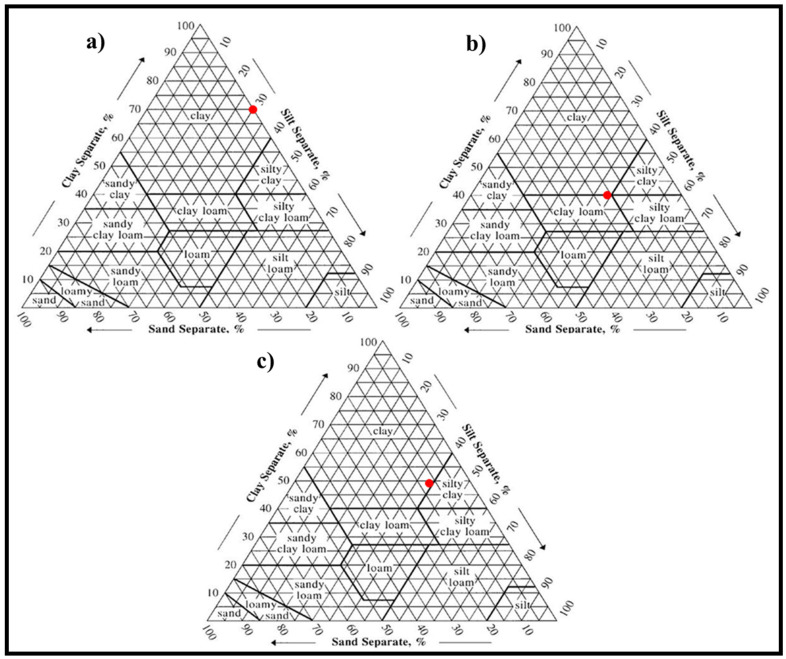
Soil texture analysis of (**a**) DM, (**b**) HM, and (**c**) SM clays.

A microgranulometric analysis using a hydrometer was carried out on the clays’ position in the soil texture diagram (Figure 2). This analysis showed that DM was composed of 69% clay-sized (0–2 µm), 29% silt-sized (2–50 µm), and 2% sand-sized (50–100 µm) fractions (Table 2). HM was composed of 40% clay-sized, 39% silt-sized (2–50 µm), and 21% sand-sized (50–100 µm) fractions (Table 2). SM was composed of 49% clay-sized, 39% silt-sized, and 12% sand-sized particles. Thus, for all the clay types, the clay-sized content of the samples was higher than the other components (silt, sand-sized particles, etc.).

**Figure 3:**
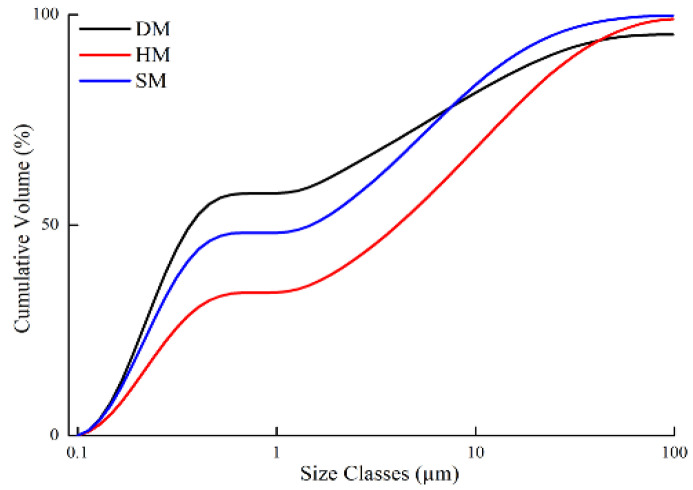
Particle size distributions of DM, HM, and SM clays.

The particle size of materials plays a significant role in plasticity. Plasticity refers to the finest fraction of the material, which is known as clay fraction (<2 μm) [40]. In ceramic brick production, attention should be focused on the finer fraction (<2 μm) of materials in ceramic brick production [41]. Thereby, the suitability of materials for clay-based brick production is enhanced. Moreover, particle size analysis was also performed using the Mastersizer Hydro 3000E (Malvern, UK) to determine its suitability for the newly established ceramics industry in Turkmenistan. The particle size analysis of DM, HM, and SM demonstrated 62.17, 39.06, and 53.6% of clay-sized fractions (<2 μm), respectively (Figure 3, Table 3). The DM showed the highest clay-sized percentage, with a 62.17% value, meaning that it was more suitable for ceramic applications [42]. The content of the clay-sized portion in the raw material is also an indicator of plasticity and workability [43]. The silt-sized content (<2 μm) of these deposits was 32.35%, 53.6%, and 44.34%, respectively (Table 3). The highest sand-sized content was in HM, at 7.34%, while it was less abundant in DM and SM (5.49% and 2.07%, respectively). The average particle size (D_10_, D_50_, and D_90_) for all clay types is also given in Table 3. The highest specific surface area (m²/kg) obtained from the Mastersizer was seen in DM (12,840 m²/kg) and SM (11,150 m²/kg) because of the clay-rich compositions. The specific surface area of clays is closely linked to particle size, and the outcomes obtained from the determination of the particle size distribution could be correlated with those of the specific surface area. According to the soil textural triangle from the particle size distribution, the DM, HM, and SM fit to the clay, silty clay loam, and silty clay region of textural composition, respectively (Table 3).

### 3.5. Dilatometric Curve

**Figure 4:**
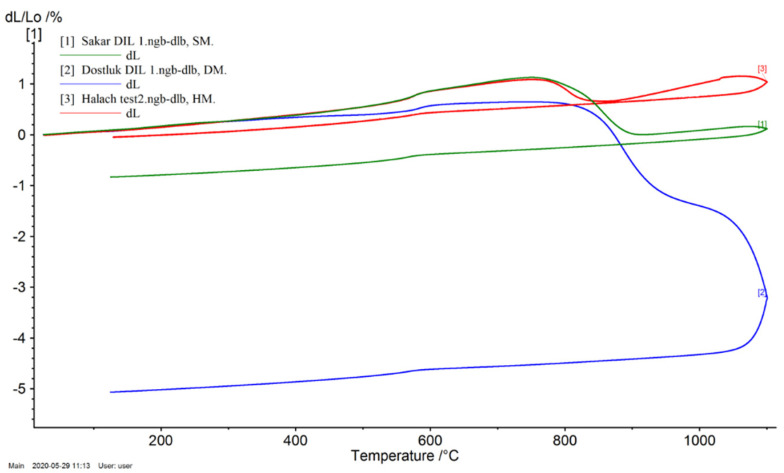
Dilatometric curves for DM, HM, and SM brick specimens fired at 1100 °C. Legends: [1] or green line, [2] or blue line and [3] or red line represents SM, DM and HM, respectively.

A dilatometric analysis was carried out to determine the shrinkage or expansion behavior of unfired specimens during firing. The dilatometric curves for DM, HM, and SM specimens at 1100 °C are shown in Figure 4. In general, these clay types presented rather similar behaviors. In all the clay types, steady and soft expansions were observed until the quartz polymorphic inversion α→β at 573 °C. Beyond this point (573 °C), the expansion rates continued to increase until they reached the maximum at 763 °C (0.51%), 753 °C (1.09%), and 758 °C (1.13%) for the DM, HM, and SM specimens, respectively. A slight shrinkage starting at over 780 °C corresponded to the formation of vitreous phases because of the illite content of the specimens. Elements, such as Fe_2_O_3_ and K_2_O alkali oxides, in the clay raw materials mainly contributed to the rapid vitrification. Considerable shrinkage of specimens occurred over 800 °C. 

The thermal expansion coefficient (TEC) values of the DM, HM, and SM clays at 300 °C and 600 °C are given in Table 4. The magnitude of thermal expansion increases with increasing temperature from 300 °C to 600 °C. While the highest TEC was observed in the HM specimens, the lowest TEC was in DM. The bigger expansions in clays are seen in the 500–600 °C zone due to the alpha–beta inversion of quartz. 

### 3.6. Thermo-Gravimetric/Differential Thermal Analysis

**Figure 5:**
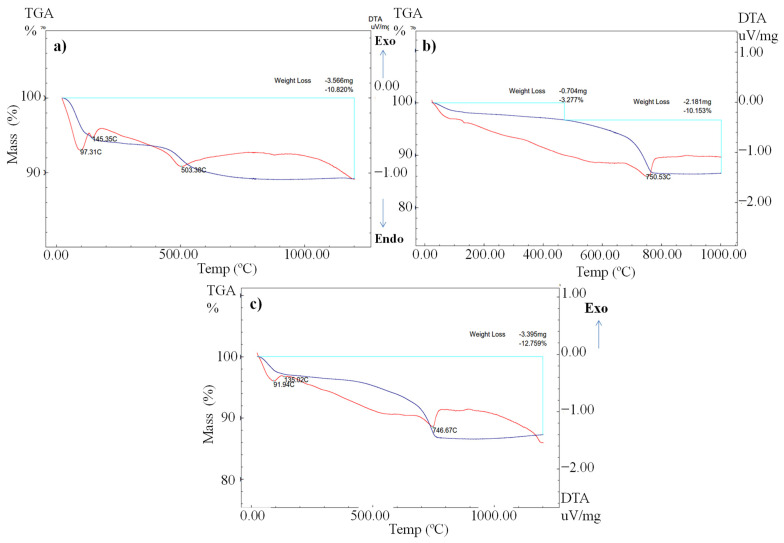
Differential thermal and thermogravimetric analysis of (**a**) DM, (**b**) HM, and (**c**) SM. Red and blue lines represent DTA and TGA diagrams, respectively.

The thermo-gravimetric analysis (TGA) of the DM, HM, and SM clays is given in Figure 5a, Figure 5b, and Figure 5c, respectively. In the thermograms, three sharp endothermic peaks occurred in all the clays. In Figure 5a–c, the differential thermal analysis (DTA) curve demonstrated a soft endothermic peak at around 97 °C, 95 °C, and 92 °C because of the elimination of hydration water [44]. The endothermic peaks attributed to mass loss around 130 °C to 150 °C are ascribed to the phenomenon of the dehydration of free water from the structure of the clays [45]. The endothermic peak at 503 °C in the DM clay corresponds to the loss of crystal water from the clay structures [46]. The existing endothermic peaks at 750 °C and 746 °C show the decomposition of CaCO_3_ and the elimination of CO_2_ in the HM and SM clays, respectively. This result is consistent with the chemical composition and mineralogical characterization of HM and SM containing CaCO_3_ in their structure. The TGA curve of the DM, HM, and SM clays demonstrated mass losses of 10.82% (3.56 mg), 10.15% (2.18 mg), and 12.76% (3.39 mg) at 1100 °C (Figure 5a–c), which is consistent with the LOI results (Table 5). Similar peaks and % weight losses were observed in the TGA and DTA thermograms of another study on illite-containing clays [47]. In that study, similar to our study, three distinct endothermic peaks were observed at 100 °C, 498 °C, and 573 °C, respectively.

### 3.7. Processing of DM, HM, and SM Clays for Brick Production

#### 3.7.1. Moisture Content

The original moisture values of DM, HM, and SM were 4.39%, 1.66%, and 2.26%, respectively. Added water percentages for these clays were 19%, 17%, and 18%, respectively (Table 5). After mixing with water, the clays were aged for one day, and the %moisture content of the humidified clays was measured as 17.03%, 16.22%, and 18.36%, respectively. These moisture content values between the range of 15 and 20% correspond to stiff extrusion values [30]. The moisture content of clay indicates the values of the samples’ porosity and linear shrinkage. Further, well-adjusted water content makes clays moldable and sinterable by enhancing their plasticity and strength.

#### 3.7.2. Plasticity Evaluation

**Figure 6:**
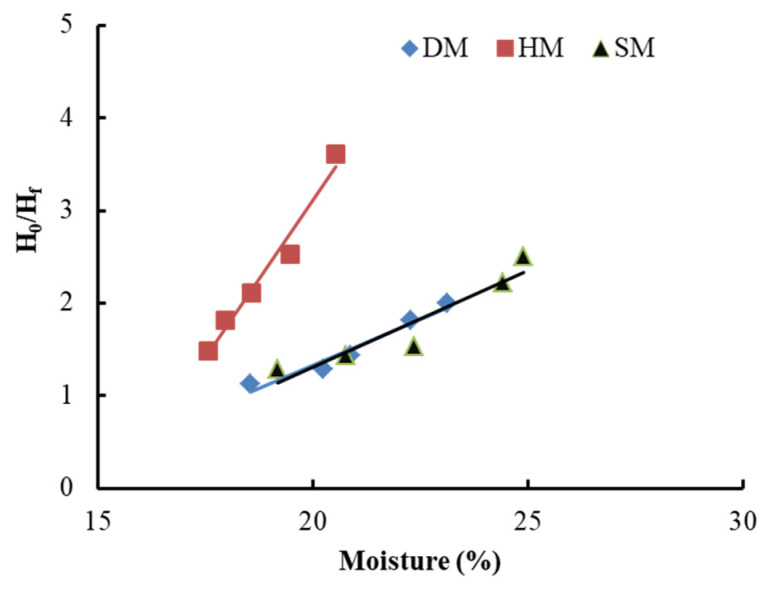
Typical chart of Pfefferkorn for DM, HM, and SM clays.

The outcomes of the PPI for the DM, HM, and SM clays are illustrated in Figure 6 and Table 5. The plasticity of these clays and their adjustability for pressing are distinctly linked. Lower plasticity means higher energy consumption and higher forming force in the processing of ceramic products. The PPIs for the DM, HM, and SM were determined to be 29.45, 19.04, and 28.74, respectively, from the Pfefferkorn straight lines. The DM and SM clays were considerably more plastic than HM, explaining their excellent aptitude for pressing (Table 5). This can be attributed mainly to the chemical and mineralogical composition of DM and SM and, to some extent, the particle size of the clays. The lowest PPI value was observed for HM clay, which showed the least plasticity due to its higher quartz content [48]. Measurements of plasticity with the penetrometer were also performed to determine the water content and penetration resistance of all the clay types. The penetrometer is considered easy to use, more coherent, less operator-dependent, and has better reproducibility [49]. The penetrometer values of DM, HM, and SM were 2.2, 2.1, and 2.3 kg/cm^2^, respectively, which are the accepted adaptability for pressing and extruding clays. Further, the plasticity (P_s_) evaluation using (Equation (4)) was carried out to compare the clays of different deposits as another approach. The highest plasticity was also seen in the DM clay, with a value of 19.12% (Table 5). 

#### 3.7.3. Extruded Bricks from DM, HM, and SM Clays

**Figure 7:**
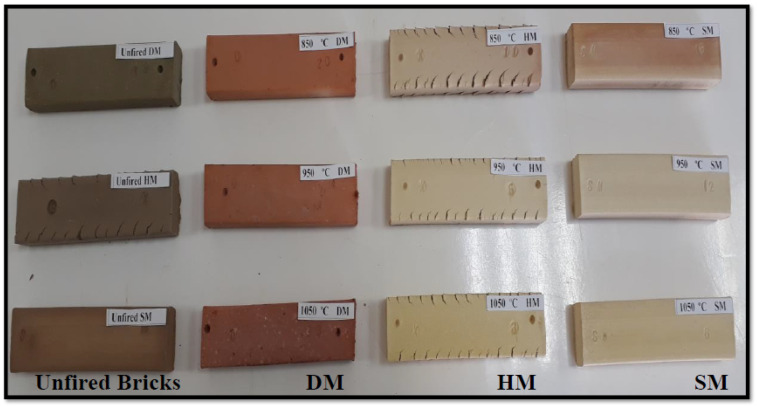
The digital images of unfired DM, HM, and SM brick specimens, and DM, HM, and SM fired at 850 °C, 950 °C, and 1050 °C.

The DM, HM, and SM clays were sprayed with a minimum amount of water (19%, 17%, and 18%, respectively) (Table 5). They were then mixed in an automatic mixer and left for ageing (a process to enhance the clay’s plasticity by storing it for a long time) to allow the clays to become fully wetted overnight. While the DM and SM bricks came out smooth, the HM bricks had cracks (dragon teeth) on the edges. The cracks of the HM bricks were caused by their higher quartz content, lower plasticity, and lower amounts of fluxes (K_2_O, Na_2_O, etc.). Although the humidity of the HM increased to some extent, the same crack formation was observed. The digital images of the extruded (unfired) DM, HM, and SM are shown in Figure 7. Despite the undesirable dragon teeth observed on the HM clays, their suitability assessment for brick production was continued, as discussed in later sections.

#### 3.7.4. Bigot’s Curves

**Figure 8:**
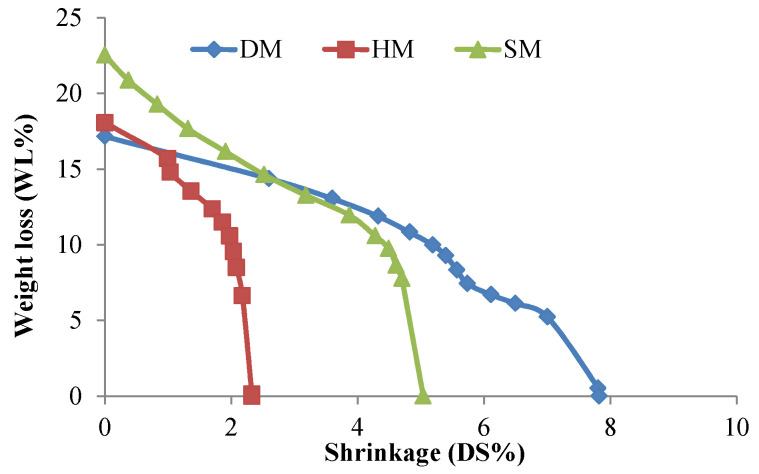
Bigot’s drying curves of DM, HM, and SM clays.

**Figure A4:**
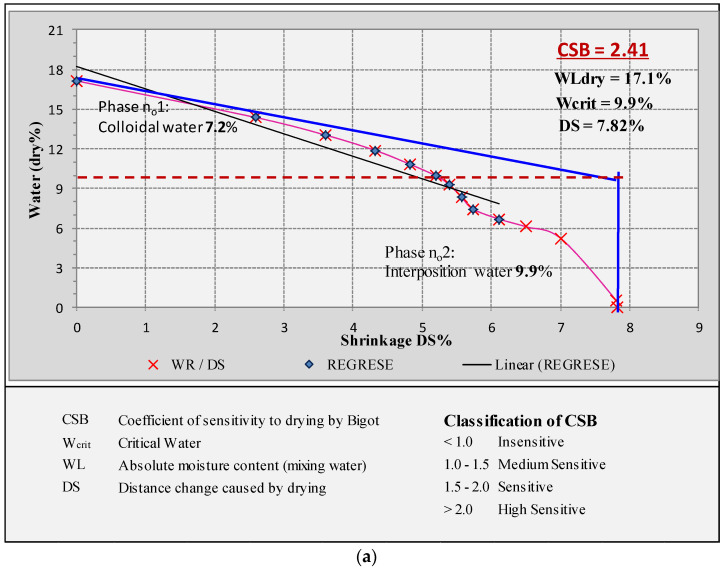

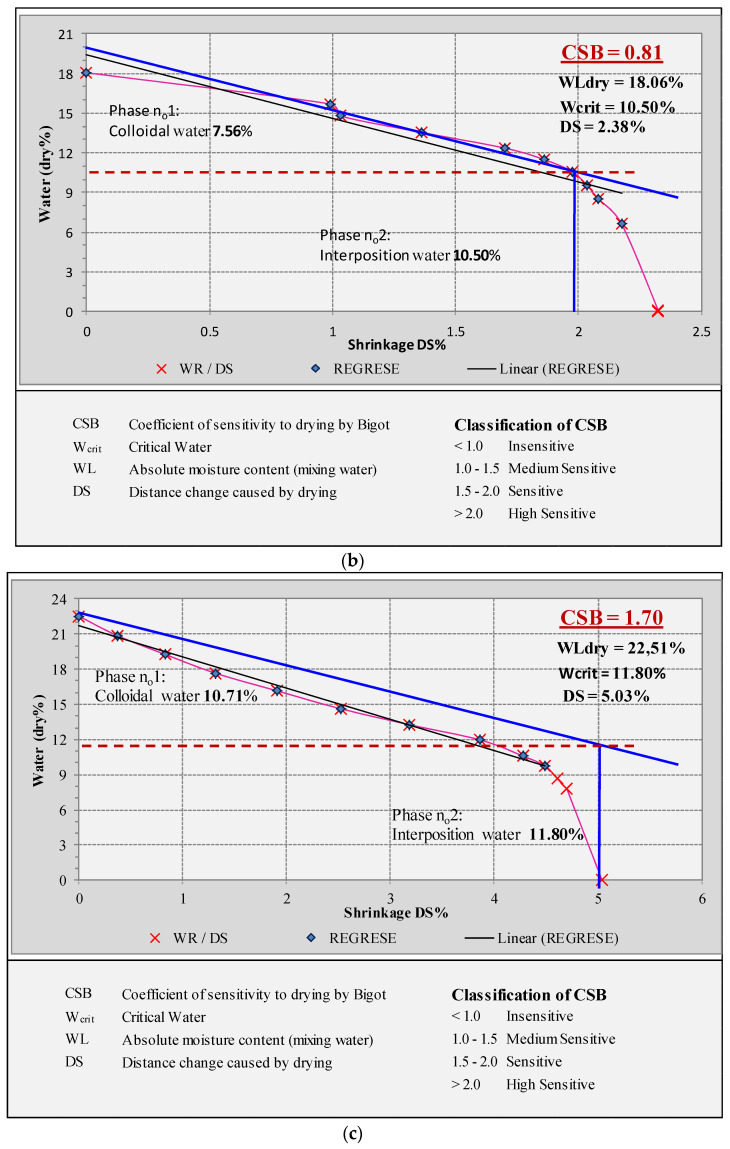
Detailed Bigot’s curves of (**a**) DM, (**b**) HM, and (**c**) SM.

Bigot’s drying curves of the DM, HM, and SM clays are presented in Figure 8. The coefficients of sensitivity to drying by Bigot (CSB) were 2.41, 0.8,1 and 1.70 for DM, HM, and SM, respectively (Figure A4, Appendix A). According to CSB classification, DM, HM, and SM fall into high sensitive, insensitive, and medium sensitive clays, respectively. The absolute moisture content (mixing water) (W_L_) values of DM, HM, and SM were 17.1%, 18.06%, and 22.51%, respectively (Figure 8). The calculated interposition water (phase number two) or critical water percentages of these clay types were 9.9%, 10.50%, and 11.80%, while the remaining percentages of W_L_ were colloidal water (phase number one). The total shrinkages (l/l′) or distance changes of DM, HM, and SM after drying were 7.82%, 2.38%, and 5.03%, respectively.

#### 3.7.5. Reabsorption of Unfired Bricks

The reabsorption of unfired bricks measures the strength of a brick’s ability to draw water from wet mortar over time. Unfired bricks absorb moisture and tend to fall apart in humid environments. Therefore, the reabsorption experiment was carried out. The reabsorption values for unfired HM, DM, and SM bricks were 5.47 ± 0.19%, 2.86 ± 0.20%, and 3.45 ± 0.14%, respectively and no degradations were observed (Table 5). The highest reabsorption was seen in DM clay because of its higher illite content.

#### 3.7.6. Production of Brick Specimens by Firing

The mineralogical, chemical, particle size, Bigot’s curve, plasticity, and technological properties of the DM, HM, and SM clays displayed promising potential for brick production. These properties determined the behaviors of the clays when molding, shaping, drying, and firing at different temperatures (850 °C, 950 °C, and 1050 °C). The extruded and shaped HM brick specimens demonstrated cracks (dragon teeth) on the edges. Although HM clay seemed unsuitable for brick production, the analysis of its suitability continued with further studies to gain insights about this clay type. This is because it could be mixed with other clays with high plasticity, making it suitable for brick production. The evaluation of the suitability of this clay for tile production continued. The firing cycles of DM, HM, and SM are given in Figure A3. The color changes resulting from firing the DM, HM, and SM bricks at different temperatures (850 °C, 950 °C, and 1050 °C) are shown in Figure 7.

#### 3.7.7. Reactions during Firing and Mineralogy of Fired Brick Specimens

**Figure 9:**
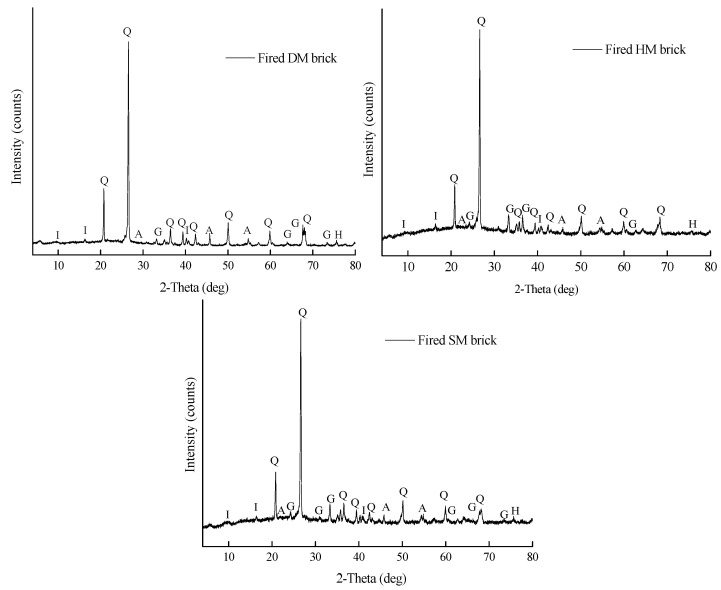
XRD spectrum of fired DM, HM, and SM brick specimens at 1050 °C.

The main stages of the firing process are evaporation, dehydration, oxidation, and vitrification, respectively [50]. In the evaporation process (20–150 °C), the free water evaporates and an endothermic reaction is observed. Secondly, in the dehydration process between 149 and 650 °C, the endothermic process takes place by the release of combined water and carbonaceous matter. Third, the exothermic reaction between 300 and 450 °C takes place as an oxidation process, oxidation of organic and subsequent sulfide compounds, followed by the endothermic reaction attributed to the conversion of mineral and quartz from α to β. Finally, the vitrification process (900–1315 °C), as an exothermic reaction, starts at about 900 °C, during which all the carbonaceous materials are completely oxidized, and then the strength of the fired bricks is improved and new crystal phases are also formed. [50]. The same endothermic peaks were also observed in the TGA spectrum of the clays given in Section 3.6. An XRD analysis of the fired brick specimens was performed to see the changes in mineralogy and the crystal structures at 1050 °C. The outcomes are demonstrated in Figure 9. After firing at 1050 °C, the illite peaks of the clays given in Figure 1 decreased because of consumption by ongoing mineral reactions. Due to the formation of a glassy phase in the HM and SM bricks fired at 1050 °C, the peak broadening was observed between 15 and 40 °2θ positions. As a primary crystalline phase, the apparent quartz (Q) mineral was identified in all the samples (DM, HM, and SM) fired at 1050 °C. Along with quartz (SiO_2_) mineral, the hematite (Fe_2_O_3_), illite (K_0.65_Al_2.0_[Al_0.65_Si_3.35_O_10_](OH)_2_), anorthite (CaAl_2_Si_2_O_8_), and gehlinite (Ca_2_Al_2_SiO_7_) minerals were also determined. Gehlenite and anorthite are formed by the combination of illite, silica, and CaCO_3_ present in the raw materials, respectively [51]. Minerals formed in fired bricks contribute to their physical and mechanical properties [52]. 

#### 3.7.8. Color and Sound Change of Bricks after Firing

The colors of the DM, HM, and SM clay-based bricks fired at 850 °C, 950 °C, and 1050 °C were examined and found to be different from each other, as shown in Table A2 (Appendix A). In most cases, the color of the bricks is an important aesthetic property for the market. The images of the unfired and fired DM, HM, and SM bricks are shown in Figure 7. While the unfired bricks are shown on the left, the fired bricks at 850 °C, 950 °C, and 1050 °C are shown on the right. The color of DM was red at 850 °C and 950 °C, becoming browner as the temperature increased because of higher iron oxide (Fe_2_O_3_) content [53]. Although SM is red clay, the red color changed after firing from reddish to dark beige because of its high carbonate content [54]. All the brick specimens for the different temperatures produced a metallic sound upon contact with a fired ceramic product or iron object.

#### 3.7.9. Drying and Firing Shrinkage

**Figure 10:**
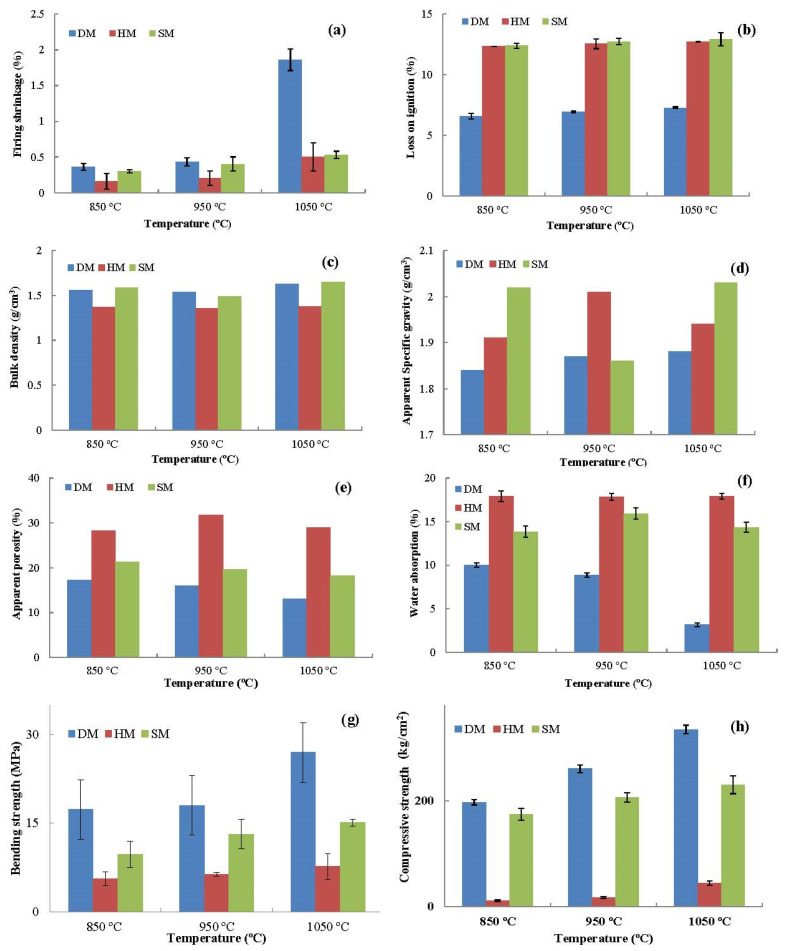
Results of the technological parameters according to the fired temperatures (850 °C, 950 °C, and 1050 °C) of DM, HM, and SM brick specimens: (**a**) firing shrinkage (%), (**b**) loss on ignition (%), (**c**) bulk density, (**d**) apparent specific gravity, (**e**) apparent porosity (%), (**f**) water absorption (%), (**g**) bending strength (MPa), (**h**) compressive strength (kg/cm^2^).

Table 5 and Figure 10a demonstrate the drying shrinkage and volume variations at 110 °C and the firing shrinkages of the DM, HM, and SM specimens (850 °C, 950 °C, and 1050 °C). The drying shrinkages of DM, HM, and SM were 6.66 ± 0.45%, 2.82 ± 0.50%, and 5.46 ± 0.78%, respectively. These values are consistent with total shrinkage values obtained by the Bigot analysis in Section 3.7.4. For all firing temperatures, the shrinkage increased as the firing temperature increased for all the clay types. The highest shrinkage was observed in the DM clay, with values of 0.36 ± 0.05%, 0.43 ± 0.05%, and 1.86 ± 0.15% at 850 °C, 950 °C, and 1050 °C firing temperatures, respectively. The lowest firing shrinkage was observed in the HM clay (0.16–0.5% at 850–1050 °C) as a result of the lowest clay content in its structure. Another reason for this outcome was the higher content of quartz in HM than in other clay types. This led to less shrinkage and adequate densification in the fired brick specimens [7]. Overall, the physical and chemical events occurred during the shrinkage process, namely decomposition, phase transformation, and sintering with partial melting [55]. The minerals in the clays underwent various processes, such as the release of water from kaolinite, quartz conversion to tridymite, and transformation to metastable cristobalite. These processes led to promising condensed mineral phases and the formation of more stable structures. Further, they initiated the formation of glassy phases as well as structural reorganization, which causes shrinkage [56]. 

#### 3.7.10. Loss on Ignition (LOI)

The LOI test demonstrated the loss of mass at high temperatures of 850 °C, 950 °C, and 1050 °C. The LOI values of the bricks fired at these temperatures are given in Table 5. An increase in temperature increased the LOI values of the brick specimens, attributed to the elimination of organic matter, hydroxides and carbonates, the oxidation of some chemical elements, or the transformation of some chemical compounds [57]. The LOI values obtained from the TGA analysis were rather similar to the LOI values at the firing temperatures of 850 °C, 950 °C, and 1050 °C. The LOI values obtained from the TGA analysis were rather similar to the LOI values at 850 °C, 950 °C, and 1050 °C (Figure 10b). The highest and lowest LOI values were observed in the DM and SM clays at 12.93 ± 0.5% and 7.27 ± 0.07% at 1050 °C, respectively (Table 5). The reason for the highest LOI for DM and SM was illitic clay, which contains a significant amount of molecular water and high soluble salt content.

#### 3.7.11. Apparent Porosity, Apparent Specific Gravity, and Bulk Density

Bulk density is defined as the weight (w) of a unit volume for fired brick specimens. In brief, it indicates the concentration of voids, micropores, holes, and cavities of fired bricks because these parameters determine the final density of fired bricks. As demonstrated in Figure 10c, the brick specimens fired at 1050 °C had slightly higher bulk densities than those fired at 850 °C and 950 °C because of the densification of microstructure, consolidation between the particles, and vitrification of specimens. These outcomes are consistent with prior studies on clay-based brick specimens [7,40]. Furthermore, the opposite correlation between apparent porosity and bulk density was also observed when these two parameters were compared.

The apparent porosity values of the DM, HM, and SM clay-based brick specimens are given in Figure 10e. The brick specimens fired at 1050 °C displayed lower apparent porosity than those fired at 950 °C in all the clay types from different origins. Such a tendency has been recorded in many studies, resulting from the increased densification of brick specimens at high temperatures, especially at T > 1000 °C [12,52]. Fluctuations in apparent porosity values between the three temperatures (850 °C, 950 °C, and 1050 °C) in the HM and SM bricks are the result of carbonate decomposition in the structure of these clays at temperatures of 800 °C to 1000 °C. These experimental outcomes show that the greatest carbonate release was observed at 950 °C. The greatest carbonate decomposition was observed at 950 °C in all carbonate-containing clay-based brick specimens. The generation of new crystalline phases upon direct reaction with carbonate or calcite in clay minerals cannot be ruled out because, in some cases (e.g., with illite), they decompose entirely only at temperatures slightly above 950 °C [51]. The reaction of the calcium carbonate decomposition occurs as follows:

CaCO_3_ ↔ CaO + CO_2_ (800–1000 °C)

After carbonate decomposition and the release of CO_2_ at 800 and 1000 °C, the apparent porosity decreased at 1050 °C. The apparent porosity has decreased due to the absence of carbonates in the structure of the clay used in the production of DM brick specimens. The decrease in porosity observed with increasing temperature and pressure is due to the coalescence of the molten phase and the powder compaction under pressure, respectively. 

Apparent specific gravity as a significant parameter is generally expressed as the ratio of the mass of a unit volume of the impermeable part of the aggregate, which means that it does not include the permeable pores in the aggregate. The AS gravities of the brick specimens are presented in Figure 10d, indicating that there are slight changes between clay types and firing temperatures. The highest and lowest AS values were obtained in SM clay fired at 1050 °C and DM clay fired at 850 °C with 2.03 and 1.84 AS values, respectively. Thus, the brick specimens fired at 1050 °C had a much more glassy phase than that of the bricks fired at 950 °C and 850 °C since this is fundamentally related to the total closed pore volume of the specimens [58].

#### 3.7.12. Water Absorption

Water absorption of fired brick specimens represents the porosity, capillaries, permeable pores, and voids in the matrix, which affect the performance of clay-based bricks [24]. The low water adsorption of clay-based bricks is regarded as durable and resistant to harsh external weather conditions [37]. According to the *ASTM C62*, the value of water absorption of less than 22% is recommended in mild weather conditions [59]. It has also been suggested by other researchers that the maximum water adsorption limit for clay-based bricks should be between 20% and 30% [37,60]. Water adsorption values of bricks fired at 850 °C, 950 °C, and 1050 °C are given in Figure 10f. The water adsorption values of DM clay brick specimens were 10 ± 0.26%, 8.86 ± 0.75%, and 3.15 ± 0.21% for 850 °C, 950 °C, and 1050 °C, respectively. There was a gradual decrease in water adsorption with increasing temperature. The HM clay bricks showed little difference between the different temperatures (850 °C, 950 °C, and 1050 °C) as 17.90 ± 0.6%, 17.83 ± 0.44%, and 17.92 ± 0.35%, respectively. The water absorption values of fired SM bricks were 13.84 ± 0.66%, 15.93 ± 0.64%, and 14.33 ± 0.57% for 850 °C, 950 °C, and 1050 °C, respectively. The increase of water absorption from 850 °C to 950 °C resulted from the presence of carbonates in the structure of SM clays. The reason for this was the formation of pores in the clay matrix of SM during the decarbonation of crystalline calcite or calcium carbonate when fired between 800 °C and 1000 °C. The DM does not contain any carbonates and has a higher clay percentage for optimal sintering of the clay minerals within voids and capillaries. The increasing temperature for firing the brick specimens reduced the water adsorption, which can be attributed to the densification of microstructure and closing pores as a result of the sintering process. Water absorption of all clay types was lower than 22%, which means that it is satisfactory for mild weather conditions. Thus, these W_A_ values demonstrate that all clays can be used in mild weather conditions, leading to sustained construction.

#### 3.7.13. Bending and Compressive Strength

The mean values of bending strength of fired brick specimens (850 °C, 950 °C, and 1050 °C) are demonstrated in Figure 10g. The bending strength values of unfired brick specimens were 6.16 ± 0.77, 2.04 ± 0.21, and 3.99 ± 0.20 Mpa for DM, HM, and SM, respectively. The bending strength of the specimens significantly increased as the temperatures increased for all clay types. The highest and lowest bending strengths were observed in DM and HM clays, respectively. The percentages of increase from 850 °C to 1050 C were 55.60%, 36.34%, and 55.09% for DM, HM, and SM, respectively. Bending strength increases with temperature because of the higher sintering degree of the brick specimens and the formation of crystalline phases through silica in the structure of clay. Further, the higher temperature increases the densification of the brick specimens through the development of a vitreous phase. Consistently, the compressive strengths of the clay types also increased as the temperature increased. The compressive strength of the unfired bricks was 44.2, 7.5, and 41 kg/cm^2^ for DM, HM, and SM, respectively (Table 5). After firing at 1050 °C, the compressive modulus increased at least four-fold compared with unfired brick specimens. The mechanical test values demonstrated that the outcomes of DM, HM, and SM were sufficient, achieving a compressive modulus of over 11 Mpa. This exceeded the 10 Mpa (minimum value) required for brick standards, which is deemed acceptable by regulations [61]. HM had the lowest compressive strength, as can be seen in Figure 10h. The compression modulus values of DM and SM-based bricks were higher than those of HM clay-based bricks. As demonstrated by this study, the firing temperatures of brick specimens have significant effects on bending and compressive strength, and it appears that DM and SM clays are suitable for brick production.

#### 3.7.14. Optic Analysis

**Figure 11:**
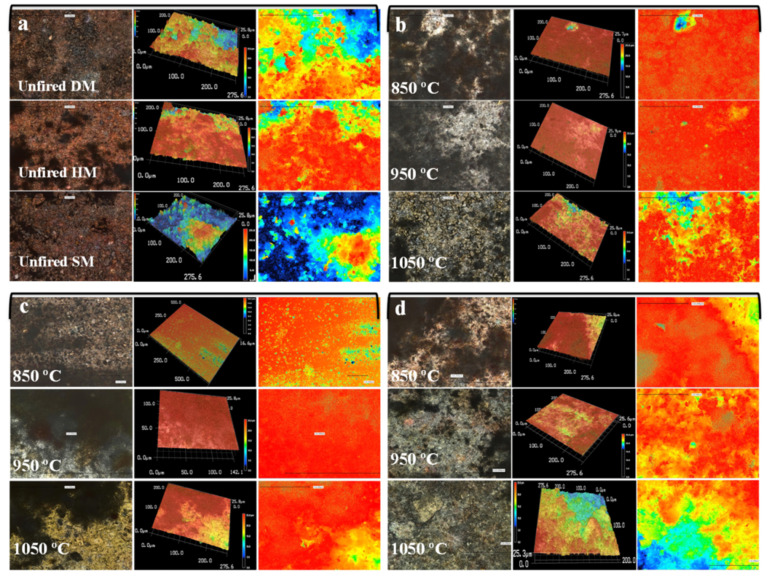
Optical images of (**a**) unfired DM, HM, and SM, fired (at 850 °C, 950 °C, and 1050 °C), (**b**) DM specimen, (**c**) HM specimen, (**d**) SM brick specimens.

The optical images of brick specimens (unfired, 850 °C, 950 °C, and 1050 °C) are shown in Figure 11. All unfired specimens presented a rougher texture. After firing at 850 °C, 950 °C, and 1050 °C, the texture of the specimens changed moderately. After firing at 850 °C, the surfaces of brick specimens became smoother than the unfired specimens. Smoother surfaces and a finer texture were obtained in DM and HM bricks fired at 950 °C. The surface of HM became smoother because of the high content of quartz. In SM bricks fired at 950 °C, the surface became slightly rougher. This may be due to the high carbonate content (12.90%) of SM clay and its decomposition between 800 C and 1000 °C [62]. At 1050 °C, the roughness increased in all clay-type bricks. The voids and cracks on the surface of specimens that appeared as dark-brownish markings were also originally present. The reason for this may be the insufficient chemical bonding of the existing particles with clay compounds. There are still some cracks and voids in the structure, and the number of these voids decreased significantly as the firing temperature increased, especially at 1050 °C. Cracks observed in brick specimens at T < 1000 °C began to disappear at 1050 °C because of extended vitrification and densification of the microstructure.

### 3.8. Scanning Electron Microscopy Analysis of Brick Specimens

**Figure 12:**
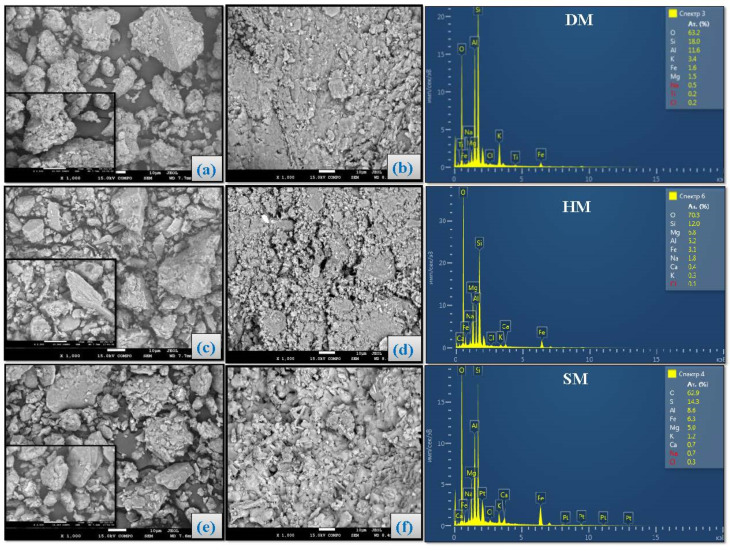
SEM images of (**a**) DM powder and (**b**) unfired DM brick, (**c**) HM powder and (**d**) unfired HM brick, (**e**) SM powder and (**f**) unfired SM brick. EDX spectrum of DM, HM, and SM are shown on the right.

Particle morphologies and elemental analysis of the DM, HM, and SM clays are shown in Figure 12. The clay particles were in the form of micron-sized agglomerates. There were no apparent variations in the sub-micron and micron structure of the DM, HM, and SM clay particles. All the clay types possessed irregular shapes with angularity and different sizes. The scanning electron microscopy (SEM) results of the extruded unfired brick specimens demonstrated the densification of microstructure that was achieved by physical and chemical effects under vacuum (Figure 12d–f). Micropores formed on the surface of the brick specimens at a range of 1–20 μm. To investigate the change of surface elements of DM, HM, and SM, the EDX spectra of DM, HM, and SM clay and unfired bricks are shown in Figure 12. As demonstrated in the EDX spectrum of DM clay, the major peaks of Si, O, Al, and Fe are evident with the atomic percent of 22.8 ± 6.78%, 64.55 ± 1.90%, 6.90 ± 6.64%, and 1.1 ± 0.70%, respectively. The chemical (oxides) and energy-dispersive X-ray (EDX) analysis showed the presence of Si, Fe, and Al contents in all clays. In addition, other peaks of metals, such as K (2.6 ± 1.13%), Mg (2.1 ± 0.98%), Ca (0.7%), Na (0.5%), and traces of Ti, Cu, S, and Cl, also existed in Figure 12a,d in all clay types. The atomic percentages of HM (Si (11.05 ± 0.95), O (67.70 ± 2.60), Al (6.10 ± 0.90), and Fe (4.15 ± 1.05)) and SM (Si (13.45 ± 0.85%), O (67.95 ± 5.05%), Al (7.75 ± 0.85%), and Fe (3.8 ± 0.85%) were also similar to DM clay. Both HM (Figure 12b,e) and SM (Figure 12c,f) were rich in Mg elements with 8.2 ± 1.14% and 3.75 ± 1.25%, respectively. The elemental analysis by EDX also confirmed the chemical analysis obtained by XRF analysis.

**Figure 13:**
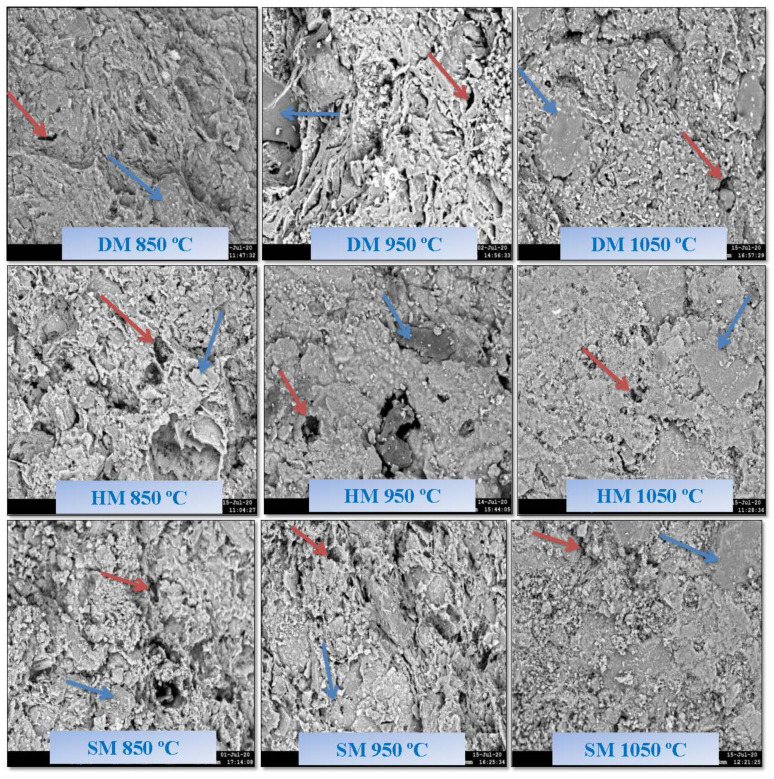
SEM micrographs of fired bricks of DM, HM, and SM at 850 °C, 950 °C, and 1000 °C, respectively.. Red arrows show pores and blue arrows show vitrification.

Figure 13 shows the SEM examinations of the fired bricks at temperatures of 850 °C, 950 °C, and 1050 °C, respectively. A dense network of fiber-like particles was observed due to the dehydroxylation of micaceous or illitic species at lower temperatures [63]. Figure 13 also shows that the brick specimens fired at 950 °C had more micropores than at 850 °C. This is because of the release of carbonates from the structure of the brick specimens. Carbonate decomposition occurs between 800 °C and 1000 °C, while color changes from reddish to yellow [55]. The increase in porosity at 950 °C could be explained by the continuing release of CO_2_ as a result of calcium carbonate decomposition [64]. The water absorption was slightly increased because of continuing carbonate release at 950 °C. Vitrification was also observed in all the brick specimens fired at 950 °C and 1050 °C. Because of the high carbonate content of SM and salt content of DM, there were signs of partial vitrification with angular morphology at 850 °C in these clay types as observed in a prior study [65]. At 1050 °C, smooth portions were formed, and the vitreous phase started to fill the pores, thereby reducing overall porosity in all clay types without carbonate release. In short, the clay particles clump together, forming featureless regions consisting mostly of a glassy phase with increasing temperature and firing time.

### 3.9. Freeze–Thaw Resistance

**Figure 14:**
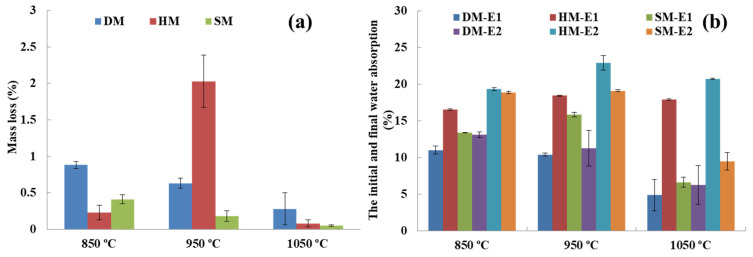
Freeze–Thaw Resistance after 200 cycles **(a)** Mass loss (%) due to freeze–thaw; (**b**) the initial and final water absorption.

Interaction between clay-based bricks as a building material and climatic factors plays an important role in the freeze–thaw resistance of bricks [66]. During the freeze–thaw cycle, water diffuses inside the pores, freezes at −5 °C, and the expansion of the brick specimens occurs. The expansion of water can occur up to 9% during the phase of liquid changing to a solid-state [67]. This process can cause damage or cracking if the pore volume of the specimens is less than the volume of the expanding water [68]. Figure 14 demonstrates the mass loss%, initial, and final water absorption (E1 and E2) of the freeze–thaw cycles for DM, HM, and SM clay-based brick specimens. After 200 cycles, the mass loss% was less than 2 for all clay types except HM. In HM fired at 950 °C, there were some cracks on the edge of the brick specimens after 159 cycles. This cracked brick specimen of HM fired at 950 °C was removed from the cycle and the mass loss value was measured as 2.03 ± 0.36%. These cracks considerably increased the mass loss% of the HM clay-based bricks. The mass loss% of all the clay types was less than 3%. Based on *ASTM C67*, brick specimens can be considered inadmissible if the mass loss increases by 3% or if it cracks during freeze–thaw cycles [26]. The initial and final water absorption of brick specimens after 200 cycles was also determined (Figure 14b). After the freeze–thaw cycles, there was an increase in water absorption of approximately 15% except for DM fired at 950 °C. The DM brick specimens fired at 950 °C increased from 10.39% to 11.27% water absorption with only an 8.40% increase. The HM clay-based bricks fired at 950 °C with water absorption of 22.91% exceeded the 22% threshold value for water absorption standards. The reason for this may be the existing cracks (dragon teeth) on the edges of the HM clay-based bricks and low mechanical properties.

### 3.10. Semi-industrial Trials of DM, HM, and SM Clay-Based Bricks

**Figure 15:**
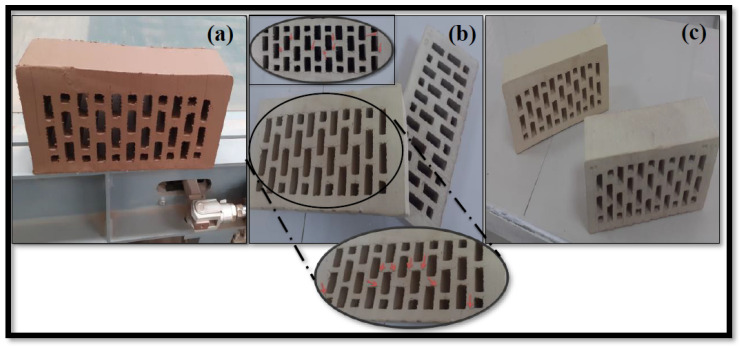
Industrial trial bricks of (**a**) DM, (**b**) HM, and (**c**) SM manufactured in the ceramic plant. Arrows showing the crack on HM bricks.

Based on the promising features of the DM and SM clays, semi-industrial trials were carried out to test clay suitability. The results were encouraging for mass production in the newly established ceramic plant in the Lebap region of Turkmenistan. The HM bricks were also tested under plant conditions. The hollow brick samples were the first step in determining whether or not laboratory outcomes would encourage a shift from the laboratory to an industrial scale. In general, the brick specimens should not contain breaks, cracks, defects, or deformations. The brick specimens obtained under plant conditions are shown in Figure 15a–c. The DM and SM brick specimens showed a promising appearance and were free of cracks or defects (Figure 15a,b). The colors of the hollow bricks were similar to the laboratory specimens. However, as expected, the HM bricks showed some cracks because of their low plasticity and low clay content. The arrows in Figure 15b show the cracks of HM bricks. The technological features of hollow bricks, which were similar to laboratory values, are summarized in Figure 10. The total shrinkage (drying + firing shrinkage) was less than 5.5%, fired at 950 °C for all specimens (Table 6). The LOI values were 5.4%, 16.4%, and 13.8% for DM, HM, and SM hollow bricks, respectively (Table 6). The water absorption values were also less than 16.5%, which is within the range of standard values (22%). The bending strengths of the hollow bricks for DM and SM were over 10 Mpa, while the value of HM was less than 10 Mpa. From a technological and industrial point of view, DM and SM bricks are suitable for the mass production of bricks.

### 3.11. Physical and Mechanical Features of the Mixtures of DM, HM, and SM with Waste Bricks

**Figure 16:**
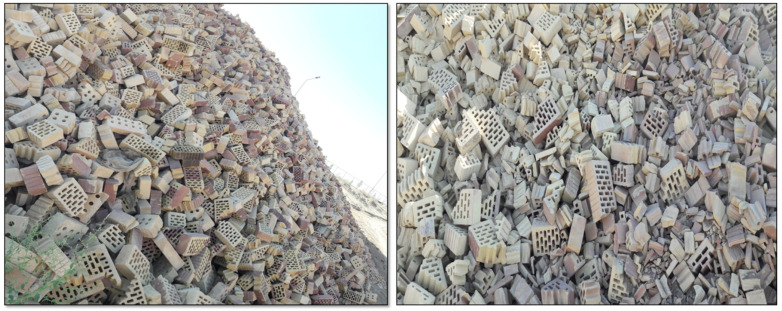
Ceramic waste bricks from the factory.

Tons of bricks leave the factory with casualties, and these waste bricks need to be recycled (Figure 16).

**Figure 17:**
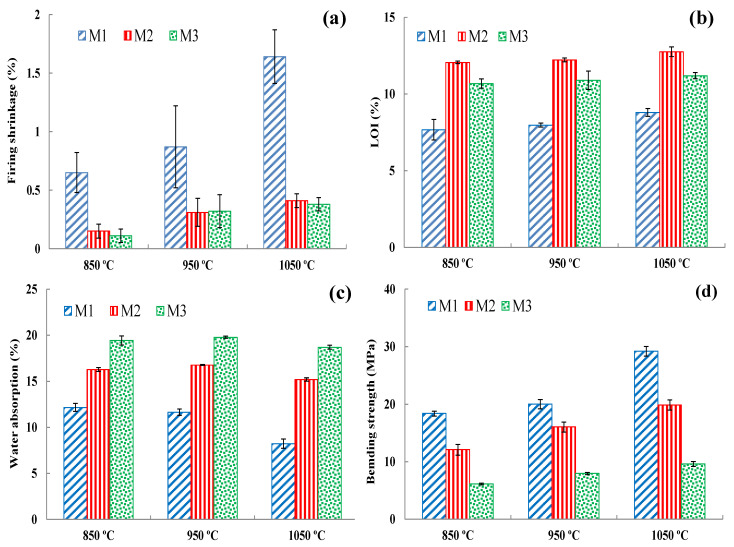
Outcomes of physical and mechanical features of fired mixtures (M1, M2, and M3): (**a**) firing shrinkage (%), (**b**) LOI%, (**c**) water absorption (%), and (**d**) bending strength (MPa).

It is necessary to include industrial waste in large quantities in the brick structure [69]. However, it is important to obtain brick samples with a smooth and homogeneous surface that does not contain fractures or cracks after extrusion. Considering these important features, the optimization studies were performed to evaluate the wastes and to add them to the clay mixtures in the maximum amount. The mixing of WB at optimum ratios with DM, HM/SM, and SM clays was performed to make mixtures of M1 (80 mass% DM + 20 mass% waste brick), M2 (85 mass% SM + 15 mass% waste brick), and M3 (70 mass% HM + 25 mass% SM + 5 mass% waste brick). With WB after extrusion, it was observed that there were no cracks at these optimum rates and the surfaces of the bricks were smoother. The outcomes of physical and mechanical features of the unfired and fired brick specimens indicate that the three mixtures exhibit a moderate change in the fired features at 850 °C, 950 °C, and 1050 °C, respectively (Figure 17). It is important to control the shrinkage with different additives, and it has been reported in the literature that it should exhibit a shrinkage below 8% for a good brick quality [70]. The drying shrinkage percentages of M1, M2, and M3 were 6.39 ± 0.12, 4.21 ± 0.08, and 5.25 ± 0.11, respectively. The firing shrinkage values decreased in all the WB-containing specimens at different ratios (Figure 17a). Figure 17d shows that the mechanical features increased in all the mixtures with the addition of the WB at all temperatures due to the densified structure of fired waste bricks compared to clay-based bricks. Densification is one of the determining parameters affecting the mechanical resistance of ceramic products [71]. Furthermore, the fired bricks contain anorthite, which helps to strengthen the mechanical properties of bricks. The same outcome was also observed in another study that the addition of waste brick in the formulation significantly improved the mechanical properties [71]. While decreases in the water absorption values were observed in M1 and M2, an increase was observed in M3 (Figure 17c). According to the *ASTM C62*, all the mixtures containing WB (water absorption < 22%) are suitable for the production of ceramic bricks [26]. The LOI values of M1, M2, and M3 were also increased with increasing temperature (Figure 17b). As a result, the usage of WB at an optimum ratio has been found to be encouraging as cost-effective alternative materials that could be utilized in fired clay-based brick production. In a very recent study in 2021, it has also been shown that the amount of clay to be used in brick-making can be reduced by up to 27% with ceramic-based waste materials [22].

## 4. Conclusions

This study examined clay deposits from three different locations in the Amu-Darya basin of Turkmenistan to determine the suitability of these raw materials for the newly developing local ceramic industry. The DM, HM, and SM clays were characterized by mineralogical, chemical, thermal, and technological analysis to assess the brick suitability for the newly established ceramic plant in Halach, Turkmenistan. The following conclusions can be drawn from the characteristics and clay-based brick production applications of illitic DM, HM, and SM clays:

- From the mineralogical analysis, illitic DM, HM, and SM clays were mainly composed of quartz, K-feldspar, and albite, respectively. X-ray spectrum analyses also revealed smectites in all three clays. Other minerals were also detected in DM (bassanite, kaolinite, chlorite, and halite), HM (kaolinite, calcite, smectite, dolomite, and chlorite), and SM (kaolinite, calcite, chlorite, smectite, dolomite, bassanite, and halite). Granulometric and size distribution analysis showed that the DM and SM clays were rich in clay-sized particles and their soil textures were clay and silty clay, respectively. Pfefferkorn analysis showed that these clay types had the highest plasticity values. HM was rich in silt-sized particles, with a prominent amount of total sand (7.34%) and quartz (33.6%). It had lower plasticity than the other clay types (DM and SM).
- The chemical analysis showed that the clays were rich in silica, which was over 50%. Other rich oxides were alumina, ferric oxide (resulting in the reddish color after firing), potassium oxide, and magnesia, respectively. The CaO from calcium carbonate is also observed in SM and HM. The lowest concentration oxides were TiO_2_, MnO, and P_2_O_3_.
- The suitability plot from chemical oxides showed that the illitic DM, HM, and SM clays could be used for producing bricks, but the HM outcome from flux agents (combined effect of Fe_2_O_3_, MgO, K_2_O, and Na_2_O) for sinterability was out of range, with an 8.90% value. The LOI values resulting from the elimination of organic matter and others for DM, HM, and SM were 7.15%, 12.40%, and 12.31% after firing at 1050 °C, respectively.
- The DM and SM clay-based bricks emerged properly from the extruder and showed high plasticity and dry-bending strength. In contrast, the HM brick specimens displayed cracks (dragon teeth) and lower plasticity and dry-bending strength.
- After firing at different temperatures (850 °C, 950 °C, and 1050 °C), all the brick specimens changed their color and sound (metallic). The bulk density, apparent density, apparent specific gravity, firing shrinkage, mass loss percentage, water absorption, freeze and thaw, bending, and compressive strengths were within the norm (standards) of building brick products. The DM and SM brick specimens had higher bending and compressive strength, while HM demonstrated the lowest strength. The strength values increased with firing temperature in all three clay types. The water absorption and freeze–thaw values of the DM and SM brick specimens were <22% and <3%, indicating that the three clays can be used for brick production. However, HM clay-based bricks displayed cracks on the edges of the bricks after 159 freeze–thaw cycles and the WA% exceeded the 22% threshold value.
- The industrial trials showed positive results for DM and SM, confirming the potential of DM as a raw material in the production of clay-based red bricks and SM as a raw material in the production of clay-based cream and dark beige bricks.
- Based on the study outcomes and observations, it can be concluded that the DM and SM clays can be effectively used for mass scale brick production, leading to energy-efficient, economical, green, and sustainable construction. HM clay could be used by mixing it with clays with high plasticity. If used alone, the brick quality will be inferior.
- Consequently, the WB can be used at optimum ratios, and the usage of WB at optimum ratios by mixing with clays will help to improve the brick features and minimize the negative impacts of their disposal.

## Figures and Tables

**Table 1:** Granulometric analysis by sieving and carbonate test results.

Sample	<80 µm		>80 µm		Total		CaCO_3_
	in gr	in %	in gr	in %	in gr	in %	in %
DM	133.98	94.43	7.91	5.57	141.89	100	1–1.5
HM	138.29	93.8	9.14	6.2	147.43	100	10.5
SM	128.29	86.71	19.66	13.29	147.95	100	12.90

**Table 2:** Mineralogical and chemical composition (oxide content %) of the DM, HM, and SM clays.

	DM (%)	HM (%)	SM (%)
Physical properties			
Clay-sized particles (<2 µm)	69	40	49
Silt-sized particles: (2–50 µm)	29	39	39
Sand-sized particles: (>50 µm)	2	21	12
Chemical composition (%)			
SiO_2_	57.39	50.20	53.09
Al_2_O_3_	16.90	14.70	12.30
Fe_2_O_3_	6.28	2.69	5.55
K_2_O	3.52	1.76	2.91
Na_2_O	1.87	1.98	2.21
MgO	2.23	2.47	2.35
TiO_2_	0.78	–	–
P_2_O_5_	0.23	0.19	0.12
CaO	2.15	12.70	8.55
MnO	0.23	0.19	0.21
LOI (1050 °C)	7.15	12.40	12.31
Total (oxides)	99.47	99.53	99.58
Total C (%)	0.12	1.73	2.08
Total S	0.3	–	0.07
Mineralogical properties			
Illite	+++++	+++	++++
Quartz	+++	++++	+++
K-Feldspar	+++	+++	+++
Albite	–	+	–
Calcite	+	++	++
Chlorite	+	+	+
Dolomite	–	+	+
Smectite	+	+	+
Kaolinite	++	++	+
Bassanite	+	–	+
Halite	+	–	+

Legends: +++++ (>30%), ++++ (>20%), +++ (>10%), ++ (>5%), + (<5%), – not detected.

**Table 3:** Physical properties of DM, HM, and SM clays.

Type	DM	HM	SM
D_10_ (µm)	0.154	4.05	0.158
D_50_ (µm)	0.369	30.7	1.48
D_90_ (µm)	22.9	-	15.3
Specific surface area (m²/kg)	12,840	7896	11,150
Clay-sized (<2 μm)	62.17	39.06	53.6
Silt-sized (2–50 μm)	32.35	53.6	44.34
Very fine sand (50–100 μm)	0.78	3.32	0.81
Fine sand (100–250 μm)	0.03	0.08	0.13
Medium sand (250–500 μm)	1.59	1.05	0.12
Coarse sand (500–1000 μm)	2.92	2.8	0
Very coarse sand (1000–2000 μm)	0.16	0.09	0
Total sand (50–2000 μm)	5.49	7.34	2.07
Soil texture	Clay	Silty clay loam	Silty clay

**Table 4:** Thermal expansion coefficients of DM, HM, and SM specimens.

Samples	Thermal Expansion Coefficient (α) 10^−6^ K^−1^	
	300 °C	600 °C
DM	8.34	18.72
HM	22.92	31.27
SM	9.71	24.34

**Table 5:** Technological features of clays and unfired brick specimens.

	DM	HM	SM
Color of clay	Greenish	Dark beige	Red
Moisture content%	19	17	18
PPI	29.45	19.04	28.74
Penetrometer consistency	2.2	2.1	2.3
Plasticity (Ps)	19.12 ± 0.23	16.30 ± 0.15	16.42 ± 0.41
Readsorption (%)	5.47 ± 0.2	2.86 ± 0.2	5.46 ± 0.78
Unfired specimen bending strength (MPa)	6.16 ± 0.77	2.04 ± 0.21	3.99 ± 0.2
Compressive strength unfired (kg/cm^2^)	44.2	7.5	41
Drying Shrinkage (%)	6.66 ± 0.45	2.04 ± 0.21	3.99 ± 0.2
LOI (%) by TGA	10.82%	10.15%	12.75%

**Table 6:** Technological features of fired hollow brick samples at 950 °C.

Samples	Drying Shrinkage (%)	Temperature (°C)	Firing Shrinkage (%)	LOI %	Water Absorption (%)	Bending Strength (MPa)
DM	3.95 ± 0.2	950	1.3 ± 0.05	5.4 ± 0.3	8.5 ± 0.3	16.43 ± 0.2
HM	2.35 ± 0.05	950	0.15 ± 0.01	16.4 ± 1.2	17.75 ± 1.0	6.07 ± 0.6
SM	3.28 ± 0.1	950	0.39 ± 0.05	13.8 ± 0.5	13.76 ± 0.7	12.9 ± 1.9

## Appendix A

**Table A1:** The concentrations of soluble salts in DM, HM, and SM clays.

Type	CO_3_^2−^		HCO_3_^−^		Cl^−^		SO_4_^2−^		Total Anion		Ca^2+^		Mg^2+^		Na^++^ K^+^		Total Cation		Total Soluble Salt%	pH
	mmol/100 g	%	mmol/100 g	%	mmol/100 g	%	mmol/100 g	%	mmol/100 g	%	mmol/100 g	%	mmol/100 g	%	mmol/100 g	%	mmol/100 g	%		
HM	0.43	-	0.55	0.034	0.55	0.02	0.69	0.033	1.79	0.086	0.55	0.011	0.30	0.004	0.940	0.022	1.79	0.036	0.13	6.8
SM	2.0	0.25	0.95	0.058	0.70	0.025	0.45	0.022	4.10	0.104	0.10	0.002	0.15	0.002	1.850	0.043	2.10	0.046	0.23	7.8
DM	0.016	0.01	0.70	0.043	2.50	0.089	2.86	0.137	6.07	1.469	1.10	0.022	0.45	0.005	4.509	0.103	6.06	0.131	0.42	7.3

**Table A2:** The colors of DM, HM, and SM bricks after firing at 850 °C, 950 °C, and 1050 °C.

	DM			HM			SM		
T (°C)	850	950	1050	850	950	1050	850	950	1050
Brick Colour	Red	Red	Brown	Light coral	Beige	Tan	Reddish	Cream	Dark beige
